# A machine learning algorithm for peripheral artery disease prognosis using biomarker data

**DOI:** 10.1016/j.isci.2024.109081

**Published:** 2024-02-01

**Authors:** Ben Li, Farah Shaikh, Abdelrahman Zamzam, Muzammil H. Syed, Rawand Abdin, Mohammad Qadura

**Affiliations:** 1 Department of Surgery, University of Toronto, Toronto, ON, Canada; 2 Division of Vascular Surgery, St. Michael’s Hospital, Unity Health Toronto, University of Toronto, Toronto, ON, Canada; 3 Institute of Medical Science, University of Toronto, Toronto, ON, Canada; 4 Temerty Centre for Artificial Intelligence Research and Education in Medicine (T-CAIREM), University of Toronto, Toronto, ON, Canada; 5 Department of Medicine, McMaster University, Hamilton, ON, Canada; 6 Li Ka Shing Knowledge Institute, St. Michael’s Hospital, Unity Health Toronto, University of Toronto, Toronto, ON, Canada

**Keywords:** Artificial intelligence, Cardiovascular medicine, Machine learning

## Abstract

Peripheral artery disease (PAD) biomarkers have been studied in isolation; however, an algorithm that considers a protein panel to inform PAD prognosis may improve predictive accuracy. Biomarker-based prediction models were developed and evaluated using a model development (n = 270) and prospective validation cohort (n = 277). Plasma concentrations of 37 proteins were measured at baseline and the patients were followed for 2 years. The primary outcome was 2-year major adverse limb event (MALE; composite of vascular intervention or major amputation). Of the 37 proteins tested, 6 were differentially expressed in patients with vs. without PAD (ADAMTS13, ICAM-1, ANGPTL3, Alpha 1-microglobulin, GDF15, and endostatin). Using 10-fold cross-validation, we developed a random forest machine learning model that accurately predicts 2-year MALE in a prospective validation cohort of PAD patients using a 6-protein panel (AUROC 0.84). This algorithm can support PAD risk stratification, informing clinical decisions on further vascular evaluation and management.

## Highlights

• Biomarker-based prognostic tools are limited in peripheral artery disease (PAD)
• A machine learning model accurately predicts 2-year PAD outcomes using 6 biomarkers
• Performance remained robust on a prospective validation cohort (AUROC 0.84)
• The model can support PAD risk stratification and inform clinical decision-making

## Graphical abstract

**Figure:**
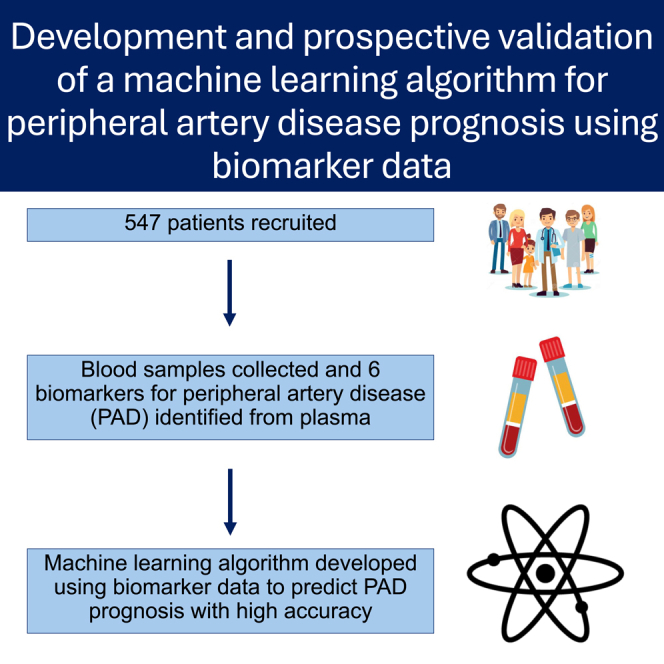

## Introduction

Peripheral artery disease (PAD) affects over 200 million people globally and manifests in claudication, rest pain, and tissue loss due to lower extremity arterial atherosclerosis.^1^ Despite its significant association with limb amputation and mortality, PAD remains poorly treated.^2^ This is partly due to a lack of biomarker-based prognostic tools to identify high-risk patients for further evaluation and treatment.^3^

Several proteins have been identified to be associated with cardiovascular diseases, including ADAMTS13,^4^ ICAM-1,^5^ ANGPTL3,^6^ alpha 1-microglobulin,^7^ GDF15,^8^ and endostatin.^9^ In fact, over 30 biomarkers for cardiovascular diseases including coronary artery disease (CAD), cerebrovascular disease (CVD), and PAD have been studied.^10^^,^^11^^,^^12^^,^^13^ The rationale for choosing these specific 37 biomarkers for analysis in this study is because they are the most widely investigated and demonstrate the strongest association with cardiovascular diseases.^10^^,^^11^^,^^12^^,^^13^ Importantly, we have designed a novel panel of proteins that have been previously studied in CAD and CVD, but not in PAD.^10^^,^^11^^,^^12^^,^^13^ Given the intricate relationship between these 3 atherosclerotic diseases, there is value in studying these biomarkers in PAD.^10^^,^^11^^,^^12^^,^^13^ Additionally, these 37 biomarkers have shown to be involved in several metabolic processes related to the development of PAD, including inflammation, atherosclerosis, thrombosis, and angiogenesis.^14^ Their mechanistic relationship to PAD supports their value as disease biomarkers.^14^ Although previous studies have demonstrated correlations between various proteins and PAD, few have characterized their prognostic value by calculating discriminatory metrics such as area under the receiver operating characteristic curve (AUROC). Furthermore, these proteins have been studied in isolation, and no previous study has investigated the prognostic value of using a combination of these proteins. Given that PAD is a multifactorial and chronic disease, with many metabolic pathways contributing to disease development,^15^ we believe that a biomarker-based protein panel, in addition to clinical features, can achieve better accuracy in predicting PAD prognosis than analyzing single proteins in isolation.

Machine learning (ML) is a rapidly evolving field that allows computers to make highly accurate predictions based on large amounts of data.^16^ Specifically, ML leverages advanced analytics to model complex relationships between inputs (e.g. biomarker levels) and outputs (e.g., PAD outcomes).^16^ Automated ML algorithms can help clinicians better understand the future clinical course of patients, augmenting the ability to provide patient-centered care with improved outcomes.^17^ This field has been driven by the explosion of clinical and biomarker-based data combined with increasing computational power.^18^ The advantage of newer ML techniques over traditional statistical methods is that they can better model complex, multicollinear relationships between covariates and outcomes,^19^ which is common in healthcare data.^20^ In a recent systematic review conducted by our group, we demonstrate that few ML-based tools for PAD prognosis consider novel biomarker data, and many suffer from high risk-of-bias, poor reporting, and inadequate performance.^21^ For example, Berger et al. (2020) developed a Bayesian model to predict 1-year hospitalization in PAD patients,^22^ and Chang et al. (2020) built a neural network to predict surgical site infection after PAD intervention.^23^ However, they did not include biomarker-based data and achieved relatively low AUROC values of 0.63 and 0.61, respectively. Therefore, additional investigation in this area is warranted. Importantly, we plan to use ML methods to link clinical to biochemical data for risk prediction, which is not heavily investigated in PAD. In this study, we used ML to develop an automated prediction tool for PAD prognosis using novel biomarker data in a propensity-score matched cohort, and real-world performance was assessed in a prospective validation cohort.

## Results

### Model development patient cohort

For ML model development, we recruited 406 patients (254 with PAD and 152 without PAD). Following propensity-score matching, we included 135 patients with PAD matched 1:1 to patients without PAD, for a total of 270 patients. In the matched cohort, the mean age was 68 (SD 10) years, 77 (29%) were female, 193 (72%) had hypertension, 205 (76%) had dyslipidemia, 79 (29%) had CAD, and 196 (73%) were taking statins. There were no differences in baseline demographic or clinical characteristics between patients with and without PAD, demonstrating appropriate matching (Table 1).

**Table 1:** Baseline demographic and clinical characteristics of propensity-score matched cohort for machine learning model development

Characteristic	Overall (n = 270)	Non-PAD (n = 135)	PAD (n = 135)	P
Age, mean (SD)	68 (10)	67 (11)	69 (10)	0.183
Female sex	77 (29)	45 (33)	32 (24)	0.899
Hypertension	193 (72)	90 (67)	103 (76)	0.080
Dyslipidemia	205 (76)	97 (72)	108 (80)	0.117
Diabetes	49 (18)	27 (20)	22 (16)	0.430
Past smoking	135 (50)	63 (47)	72 (53)	0.272
Current smoking	57 (21)	27 (20)	30 (22)	
Congestive heart failure	5 (2)	2 (2)	3 (2)	0.652
Coronary artery disease	79 (29)	36 (27)	43 (32)	0.349
Previous stroke	37 (14)	16 (12)	21 (16)	0.376
Statin	196 (73)	92 (68)	104 (77)	0.102

∗Values reported as N (%) unless otherwise indicated.

Abbreviations: PAD (peripheral artery disease).

### Protein levels

From an initial panel of 37 proteins, we identified 6 proteins that were differentially expressed in patients with vs. without PAD. Plasma concentrations of 3 proteins were lower in patients with PAD compared to those without PAD: ADAMTS13 (8.50 [SD 5.47] vs. 11.75 [SD 5.58] pg/mL, p = 0.025), ICAM-1 (65.42 [SD 5.40] vs. 80.10 [SD 6.71] pg/mL, p = 0.029), and ANGPTL3 (3.65 [SD 2.25] vs. 5.14 [SD 2.21] pg/mL, p = 0.037). Conversely, plasma concentrations of 3 proteins were higher in patients with PAD compared to those without PAD: Alpha 1-microglobulin (16.54 [SD 7.50] vs. 14.86 [SD 6.62], GDF15 (1632.29 [SD 1252.85] vs. 1164.69 [SD 794.99] pg/mL, p < 0.001), and endostatin (7.51 [SD 5.22] vs. 5.22 [SD 3.01] pg/mL, p = 0.044) (Table 2).

**Table 2:** Protein levels in patients with and without peripheral artery disease

Protein	Non-PAD (n = 135)		PAD (n = 135)		P
	Mean	SD	Mean	SD	
GDF15 (pg/mL)	1164.69	794.99	1632.29	1252.85	<0.001
ADAMTS13 (pg/mL)	11.75	5.58	8.5	5.47	0.025
ICAM-1 (pg/mL)	80.1	6.71	65.42	5.4	0.029
Alpha 1-Microglobulin (pg/mL)	14.86	6.62	16.54	7.5	0.034
ANGPTL3 (pg/mL)	5.14	2.21	3.65	2.25	0.037
Endostatin (pg/mL)	5.22	3.01	7.51	5.22	0.044
ESM-1 (pg/mL)	624.68	321.09	557.66	274.43	0.067
Chemerin (pg/mL)	11.98	4.16	11.35	1.45	0.069
Galectin-1 (pg/mL)	4.19	1.72	4.59	1.92	0.071
EpCAM/TROP1 (pg/mL)	946.77	888.63	1135.98	849.48	0.076
Tie-2 (pg/mL)	1.66	8.99	14.99	8.53	0.129
CXCL1 (pg/mL)	226.48	122.23	260.88	208.41	0.145
IL-2 (pg/mL)	67.45	32.05	73.31	35.49	0.157
BMP-2 (pg/mL)	41.66	13.81	39.17	15.77	0.171
ALCAM/CD166 (pg/mL)	1.21	4.82	1.29	6.19	0.232
Endoglin/CD105 (pg/mL)	3317.48	2634.6	2982.59	2172.52	0.258
APRIL/TNFSF13 (pg/mL)	525.24	399.36	588.36	552.94	0.284
PCSK9 (pg/mL)	1.63	2.77	1.35	1.25	0.296
CD40 (pg/mL)	439.99	245.34	465.61	241.42	0.389
CXCL6 (pg/mL)	257.46	230.75	236.62	185.56	0.415
ANGPTL6 (pg/mL)	3.66	2.33	3.43	2.26	0.421
CD62P (pg/mL)	3.34	1.86	3.2	1.19	0.449
Cathepsin S (pg/mL)	3596.23	1832.28	3757.97	1678.04	0.452
Aggrecan (pg/mL)	2.24	2.34	2.54	2.99	0.519
KIM-1 (pg/mL)	99.11	72.85	105.08	94.02	0.584
PBEF/Visfatin (pg/mL)	3.65	9.15	3.06	8.54	0.585
TNF RI (pg/mL)	1.58	1.08	1.65	0.93	0.639
Tpo (pg/mL)	1507.56	650.23	1533.34	665.87	0.748
RAGE (pg/mL)	2426.08	1359.54	2375.22	1295.02	0.754
SCF (pg/mL)	106.56	46.31	108.2	48.41	0.777
Angiopoietin-1 (pg/mL)	1.03	1.05	1.07	1.22	0.782
TNF RII (pg/mL)	3.05	2.22	2.99	1.94	0.835
IL-33 (pg/mL)	27.73	34.7	27.17	14.56	0.862
TNFRSF9/CD137 (pg/mL)	79.24	90.32	77.79	53.78	0.874
uPAR (pg/mL)	1.88	1.09	1.86	0.97	0.889
TSG-14 (pg/mL)	1289.68	585	1300.53	680.17	0.889
CD40 Ligand (pg/mL)	2.18	1.77	2.43	1.9	0.914

∗These proteins were chosen for analysis based on their involvement in various metabolic processes associated with peripheral artery disease: inflammation [interleukin (IL)-33, IL-2, urokinase plasminogen activator receptor (uPAR), chemokine ligand (CXCL) 1 and 6, thyroid peroxidase (TPO), tumor necrosis factor (TNF) receptor (R) I and II, cluster of differentiation (CD) 40 and CD40 ligand, TNF receptor superfamily (RSF) 9/CD137, galectin-1, cathepsin S, a proliferation-inducing ligand (APRIL)/TNFSF13, activated leukocyte cell adhesion molecule {ALCAM/CD166), epithelial cell adhesion molecule (EpCAM/TROP1), alpha 1-microglobulin], endothelial dysfunction [endothelial cell-specific molecule (ESM)-1, TNF-stimulated gene (TSG)-14, P-selectin (CD62P), tyrosine-protein kinase receptor (Tie-2), angiopoietin-1], angiogenesis [stem cell factor (SCF), bone morphogenetic protein (BMP)-2, cytokine nicotinamide phosphoribosyltransferase (PBEF/visfatin), endoglin/CD105, endostatin], atherosclerosis [proprotein convertase subtilisin/kexin type 9 (PCSK9), angiopoietin-like protein 3 (ANGPTL3), aggrecan, growth differentiation factor (GDF) 15], metabolic syndrome [receptor for advanced glycation end proteins (RAGE), ANGPTL6, chemerin], thrombosis [a disintegrin and metalloproteinase with a thrombospondin type 1 motif, member 13 (ADAMTS13), intercellular adhesion molecule (ICAM)-1], and renal dysfunction [kidney injury molecule (KIM)-1]. Bolded p value represents statistical significance (p < 0.05).

### PAD-related adverse events

All adverse events occurred in patients with PAD. In the PAD cohort, 42 (31%) patients developed major adverse limb events (MALE), 39 (29%) required vascular intervention, 6 (4%) underwent major amputation, and 21 (16%) had worsening PAD status over a 2-year follow-up period. The low major amputation rate likely reflects the fact that these patients were followed closely by vascular surgeons and underwent early revascularization to prevent limb loss. Therefore, the ML algorithm was not trained to predict this secondary outcome (Table 3).

**Table 3:** PAD-related adverse events over two years in propensity-score matched cohort for machine learning model development

	Overall (n = 270)	Non-PAD (n = 135)	PAD (n = 135)	P
MALE	42 (16)	0 (0)	42 (31)	<0.001
Vascular intervention	39 (14)	0 (0)	39 (29)	<0.001
Major amputation	6 (2)	0 (0)	6 (4)	0.013
Worsening PAD status	21 (8)	0 (0)	21 (16)	<0.001

∗Values reported as N (%) unless otherwise indicated.

Abbreviations: PAD (peripheral artery disease), MALE (major adverse limb event).

### Model performance

**Figure 1:**
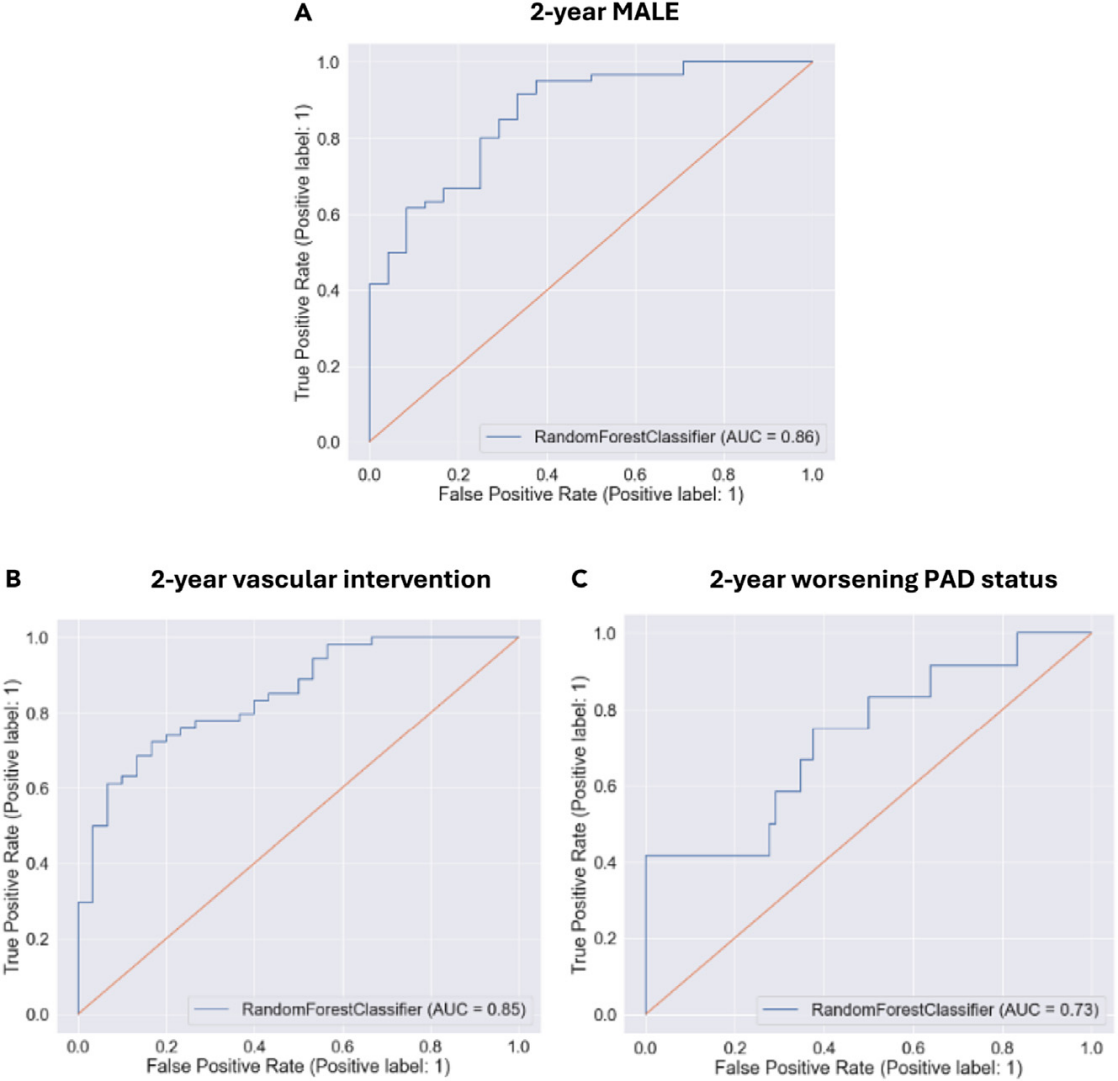
Receiver operating characteristic curve for random forest machine learning model in predicting (A) 2-year major adverse limb event (MALE), (B) 2-year vascular intervention, and (C) 2-year worsening peripheral artery disease (PAD) status on test set data AUC (area under the receiver operating characteristic curve). ∗Note: the prognostic models were built on PAD patients only because all adverse events occurred in the PAD cohort. This figure shows prognostic performance on PAD patients only.

**Figure 2:**
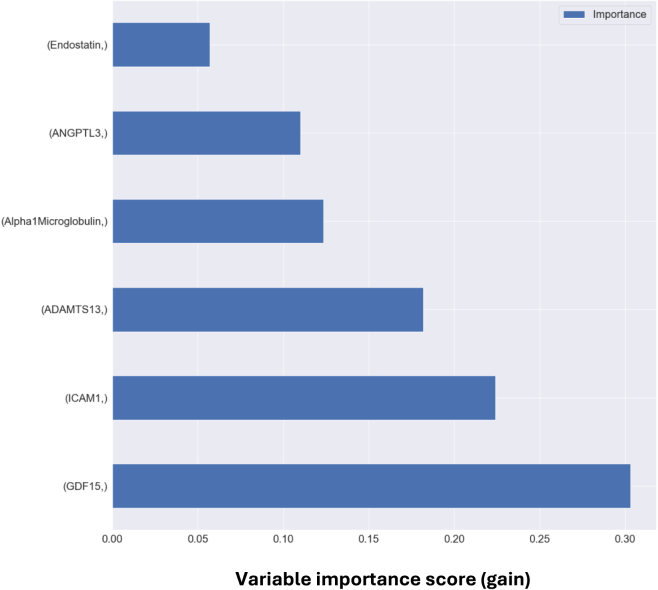
Variable importance scores (gain) for the 6 proteins used as input features for random forest machine learning model ∗Note: higher score indicates greater importance in contributing to an overall prediction.

A random forest ML model was developed with the 6 proteins identified to be differentially expressed in patients with vs. without PAD as input features. For prognosis of patients with PAD, the model achieved the following performance metrics: 2-year MALE (AUROC 0.86, Figure 1A), 2-year need for vascular intervention (AUROC 0.85, Figure 1B), and 2-year worsening PAD status (AUROC 0.73, Figure 1C). All 6 proteins contributed to model predictions with the 3 most important features being (1) GDF15, (2) ICAM-1, and (3) ADAMTS13 (Figure 2).

### Prospective validation

**Figure 3:**
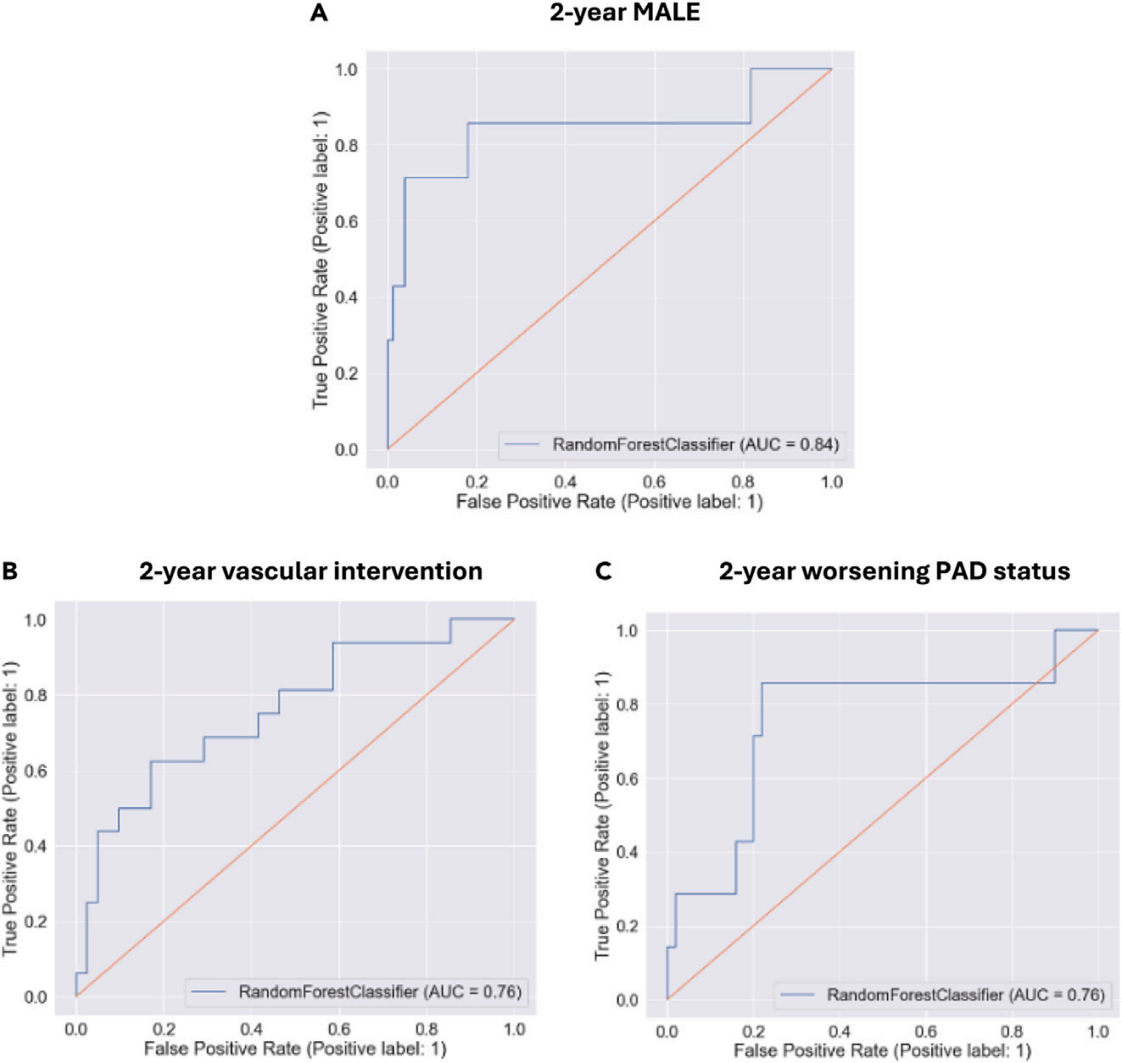
Receiver operating characteristic curve for random forest machine learning model in predicting (A) 2-year major adverse limb event (MALE), (B) 2-year vascular intervention, and (C) 2-year worsening peripheral artery disease (PAD) status in prospective validation cohort AUC (area under the receiver operating characteristic curve). ∗Note: the prognostic models were built on PAD patients only because all adverse events occurred in the PAD cohort. This figure shows prognostic performance on PAD patients only.

To validate our findings in a generalizable real-world patient population, a second prospective validation cohort was recruited. In this study phase, we included 189 patients with PAD and 88 patients without PAD, for a total of 277 patients. Compared to individuals without PAD, those with PAD were older (mean age 71 [SD 11] vs. 59 [SD 12] years, p < 0.001), more likely to have hypertension (90% vs. 33%, p < 0.001), dyslipidemia (84% vs. 35%, p < 0.001), diabetes (65% vs. 6%, p < 0.001), CAD (39% vs. 10%, p < 0.001), previous stroke (20% vs. 2%, p < 0.001), be past or current smokers (86% vs. 52%, p < 0.001), and receive statins (78% vs. 31%, p < 0.001) (Table 4). All adverse events occurred in PAD patients. In the PAD cohort, 46 (24%) developed MALE, 42 (22%) required vascular intervention, 14 (7%) underwent major amputation, and 31 (16%) had worsening PAD status over a 2-year follow-up period (Table 5). The random forest ML model built previously with the addition of clinical data as input features achieved the following performance metrics for predicting: 2-year MALE (AUROC 0.84, Figure 3A), 2-year vascular intervention (AUROC 0.76, Figure 3B), and 2-year worsening PAD status (AUROC 0.76, Figure 3C). Of note, the prognostic performance metrics reported thus far are for PAD patients only as all adverse events occurred in PAD patients. To assess generalizability of our model to the general population, we assessed prognostic performance on PAD and non-PAD patients. Our model maintained good performance, with AUROC’s of 0.83–0.88 in test set data and 0.73–0.79 on the prospective validation cohort for predicting 2-year MALE, vascular intervention, and worsening PAD status in both PAD and non-PAD patients (Figures S1 and S2).

**Table 4:** Baseline demographic and clinical characteristics of prospective validation cohort

Characteristic	Overall (n = 277)	Non-PAD (n = 88)	PAD (n = 189)	P
Age, mean (SD)	67 (13)	59 (12)	71 (11)	<0.001
Female sex	86 (31)	32 (36)	54 (29)	0.192
Hypertension	199 (72)	29 (33)	170 (90)	<0.001
Dyslipidemia	190 (69)	31 (35)	159 (84)	<0.001
Diabetes	127 (46)	5 (6)	122 (65)	<0.001
Past smoking	140 (51)	31 (35)	109 (58)	<0.001
Current smoking	68 (25)	15 (17)	53 (28)	
Congestive heart failure	11 (4)	2 (2)	9 (5)	0.323
Coronary artery disease	82 (30)	9 (10)	73 (39)	<0.001
Stroke	39 (14)	2 (2)	37 (20)	<0.001
Statin	175 (63)	27 (31)	148 (78)	<0.001

∗Values reported as N (%) unless otherwise indicated.

Abbreviations: PAD (peripheral artery disease).

**Table 5:** PAD-related adverse events over two years in prospective validation cohort

	Overall (n = 277)	Non-PAD (n = 88)	PAD (n = 189)	P
MALE	46 (17)	0 (0)	46 (24)	<0.001
Vascular intervention	42 (15)	0 (0)	42 (22)	<0.001
Major amputation	14 (5)	0 (0)	14 (7)	0.009
Worsening PAD status	31 (11)	0 (0)	31 (16)	<0.001

∗Values reported as N (%) unless otherwise indicated.

Abbreviations: PAD (peripheral artery disease), MALE (major adverse limb event).

### Supplemental analysis of diagnostic performance

The ML model achieved an AUROC of 0.87 in identifying patients with a diagnosis of PAD. Diagnostic performance remained robust on the prospective validation cohort with an AUROC of 0.85 (Figure S3).

## Discussion

### Summary of findings

In this study, we developed a robust ML model using a panel of 6 biomarkers that accurately predicts PAD prognosis in a prospective validation cohort. We showed several key findings. First, from the 37 proteins analyzed, we found 6 to be differentially expressed in patients with vs. without PAD (ADAMTS13, ICAM-1, ANGPTL3, alpha 1-microglobulin, GDF15, and endostatin). Second, we used ML-based predictive modeling to understand the collective impact of these 6 proteins on PAD prognosis. Our random forest ML model achieved excellent predictive ability for 2-year MALE, vascular intervention, and worsening PAD status in both the model development test cohort and prospective validation cohort, achieving AUROCs of 0.73–0.87. Finally, the top 3 predictive features in our algorithm were GDF15, ICAM1, and ADAMTS13. These proteins are most closely related to PAD, thereby identifying potential areas for future research to further characterize the biological relationships between these proteins and PAD development/progression.

### Comparison to existing literature

Ross and colleagues (2019) used ML technology to predict major adverse cardiac and cerebrovascular events (MACCE) in PAD patients using data from electronic health records.^24^ Using retrospectively collected International Classification of Diseases (ICD)-9 codes, Common Procedural Terminology (CPT) codes, and prescriptions, among other clinical data, the authors developed models that predict MACCE that occurs at least 30 days after PAD diagnosis, achieving an AUROC of 0.81.^24^ There were several limitations to this study. First, the model was not tested on a prospective validation cohort; therefore, its real-world performance remains unclear as developing and testing a model on the same dataset can artificially elevate performance.^25^ Second, biomarker-based data were not considered as input features, and this information can have an important impact on PAD prognosis given previously demonstrated mechanistic relationships between several biologically active proteins and PAD development.^10^^,^^11^^,^^14^ Our study addressed both limitations with consideration of biomarker data as input features in our ML models and performance evaluation on a prospective validation cohort. In our model development test set data, we achieved better performance metrics for PAD prognosis, with AUROC’s for predicting 2-year MALE above the 0.81 reported by Ross and colleagues.^24^ Expectedly, performance declined slightly in the prospective validation cohort, ranging from 0.76–0.85. Therefore, we demonstrate the value of building ML models using biomarker information, which can improve predictive performance. Furthermore, assessing performance on prospective validation datasets can provide a better understanding of real-world model performance.

Al-Ramini and colleagues (2022) used gait measurements obtained by a camera system to diagnose PAD and obtained a similar accuracy of 87%.^26^ Some of these gait measurements could also be used to quantify treatment effectiveness.^27^ More recently, others have shown that a wearable accelerometer to estimate gait can provide similarly accurate measurements.^28^ In our study, rather than using gait data, we used biomarker data to predict PAD-related adverse events. It is important to note the cost and expertise needed to run these tests, particularly given that the analyzed biomarkers remain primarily used in the research setting. Therefore, additional research is needed to demonstrate the value of these biomarkers for routine clinical use. Furthermore, future work combining gait and biomarker data using ML techniques may allow for the development of increasingly accurate prediction models for patients with PAD.

### Explanation of findings

There are several potential explanations for our findings. First, as of the 37 proteins analyzed, 6 were differentially expressed between patients with and without PAD. These proteins are involved in various mechanistic pathways important for PAD development and progression, including thrombosis (ADAMTS13^4^ and ICAM-1^5^), atherosclerosis (GDF15^8^ and ANGPTL3^6^), inflammation (alpha 1-microglobulin^7^), and angiogenesis (endostatin^9^). In our ML model, the most important predictive feature was plasma GDF15 levels. GDF15 (growth differentiation factor 15) is a protein found in circulation that promotes early plaque formation and progression.^29^^,^^30^ Mechanistically, GDF15 interacts with C–C motif chemokine receptor 2 to modulate macrophage chemotaxis, thereby downregulating macrophage apoptosis and inhibiting the proliferation of endothelial cells.^31^^,^^32^ Given that macrophage accumulation and endothelial dysfunction contribute to atherosclerosis,^33^ GDF15 plays an important role in PAD development and progression. Interestingly, de Jager et al. (2011) demonstrated that deletion of GDF15 in murine models had a beneficial effect in both early and late atherosclerosis development, indicating the importance of this protein in plaque development and progression.^29^ Clinically, De Haan and colleagues (2017) showed that higher GDF15 levels were associated with increased risk of major amputation and death in PAD patients.^8^ We similarly demonstrated the importance of GDF15 in PAD prognosis in our ML algorithm based on a biomarker panel. Although GDF15 was the most important predictive feature, the other five biomarkers also made important contributions to overall predictions based on variable importance scores. This suggests that using a panel of biomarkers likely improves accuracy in predicting PAD prognosis compared to a single protein alone, given their involvement in various biological pathways of PAD development. Second, we found high rates of adverse limb-related complications in patients with PAD, including over 20% of our cohort developing MALE. This suggests the need for more aggressive medical and possibly surgical management strategies to prevent complications in this high-risk population. Third, our ML model performed well for several reasons. Traditional statistical techniques such as logistic regression assume a linear correlation between the independent variables and the logit of the dependent variable; while advanced ML technology is not restricted by this assumption and can better model complex non-linear relationships between inputs and outputs.^34^^,^^35^ This is of particular importance in healthcare data, where a patient’s clinical outcome can be influenced by many factors.^36^ Given the advantages of ML, including automation, understanding of non-linear relationships, and accurate predictions, this technology will likely outperform traditional statistical techniques in risk prediction.^34^^,^^35^ This is particularly important in biomarker-based models, where different proteins are involved in different biological pathways and may interact in complex ways to contribute to a disease process.^37^ In our study, random forest likely achieved excellent performance because it is an ensemble learning technique consisting of the aggregation of many decision trees, which (1) reduces variance, (2) handles large datasets efficiently, and (3) reduces overfitting.^38^ Overall, we demonstrate the advantage of using a synergistic ML-based model that considers a panel of biomarkers, which likely provides better predictive performance than individual biomarker information alone. Given that PAD is a chronic and multifactorial disease involving multiple biological pathways, previous studies have suggested the value of a panel-based approach to improve the prognosis of PAD.^39^ Our study confirms that applying ML techniques to clinical data in addition to biomarkers involved in atherosclerosis, inflammation, thrombosis, and angiogenesis allows for the development of highly accurate risk prediction tools for PAD.

### Implications

Our ML models can be used to guide clinical decision-making in several ways. Our tool can be used to screen patients for asymptomatic PAD. This may be particularly useful in family practice settings, whereby generalists can send a 6-protein plasma panel in addition to routine blood work and use our automated algorithm to understand a patient’s PAD risk.^40^ Patients who screen positive for a PAD diagnosis should be sent for additional vascular evaluation, such as an arterial duplex ultrasound to assess blood flow and confirm a PAD diagnosis.^41^ Once a PAD diagnosis is confirmed, the ML algorithm can also be used with the same 6 proteins to determine a patient’s risk of adverse PAD-related events. Patients at low risk can continue receiving care from a family physician with risk factor optimization including acetylsalicylic acid, statins, and lifestyle modifications.^42^ Those at high risk of MALE or worsening PAD status should be referred to a vascular surgeon for further evaluation and management.^43^ Once a PAD referral has been made, vascular surgeons can use the ML algorithm in addition to their clinical judgment to identify those at higher risk of adverse limb events who may be considered for (1) additional vascular imaging to delineate anatomy and disease severity,^44^ (2) medical management with low-dose rivaroxaban,^45^ and/or (3) interventions for limb salvage in the highest risk patients.^46^^,^^47^ Overall, our automated ML tool can improve care for PAD patients in several ways in both the generalist and specialist settings, including providing efficient PAD screening and stratification of risk, supporting early identification and treatment of patients at high risk for adverse limb events, and reducing the number of unneeded specialist referrals, ultimately improving PAD outcomes and reducing health care costs.^48^

### Conclusions

In this study, we used a panel of 6 biomarkers to develop an ML model that accurately predicts PAD prognosis in a prospective validation cohort. Our model can be used for PAD screening and risk stratification, thereby improving early identification and targeted management of PAD. Specifically, high-risk patients should be referred for further vascular evaluation and/or receive aggressive medical management. Our ML algorithms also benefit from continuous learning and automation. This tool has the potential for important utility in the care of PAD patients. Our findings also provide insights for future research. Particularly, from a panel of 37 proteins, GDF15 was identified as the most important predictive feature for PAD prognosis. Future basic and translational studies investigating the mechanistic relationship between GDF15 and PAD development/progression may improve our understanding of pathogenesis and potential targeted therapies. Importantly, our study provides impetus for clinical trials evaluating the impact of ML algorithms on PAD outcomes.

### Limitations of the study

Our study has several limitations. First, this was a single center study using 2 recruited cohorts, and future validation at other institutions is needed to demonstrate generalizability of our model. Second, we achieved good predictive ability with a relatively small sample size for an ML-based study and future studies with larger sample sizes may improve performance. Third, 2-year outcomes are reported, and longer follow-up is needed to better understand the prognostic value of our algorithm given the long-standing nature of PAD. Fourth, the analyzed biomarkers remain primarily used in the research setting. Therefore, additional translational research and implementation science are needed to demonstrate the value of these biomarkers for routine care to support clinical use. Fifth, the ML algorithm was not trained to predict major amputation due to the low event rate, likely because patients in this study were followed closely by vascular surgeons and underwent early revascularization to prevent limb loss. Future studies are needed to assess the generalizability of the model to real-world clinical practice.

## STAR★Methods

### Key resources table

**Table:** 

REAGENT or RESOURCE	SOURCE	IDENTIFIER
**Biological samples**		

Plasma isolated from blood.	Humans.	N/A.

**Critical commercial assays**		

LUMINEX assay.	Bio-Techne, Minneapolis, MN, USA.	N/A.
Fluidics Verification and Calibration bead kits.	Luminex Corp; Austin, Texas.	N/A.

**Software and algorithms**		

MagPix analyzer.	Luminex Corp; Austin, Texas.	N/A.
Luminex xPonent software.	Luminex Corp; Austin, Texas.	N/A.
SPSS software version 23.	SPSS Inc., Chicago, Illinois, USA.	N/A.

### Resource availability

#### Lead contact

Further information and requests for resources and reagents should be directed to and will be fulfilled by the lead contact, Mohammad Qadura (mohammad.qadura@utoronto.ca).

#### Materials availability

This study did not generate new unique reagents.

#### Data and code availability

• All data reported in this paper will be shared by the lead contact upon request
• This paper does not report original code
• Any additional information required to reanalyze the data reported in this paper is available from the lead contact upon request.

### Experimental model and study participant details

#### Ethics approval

The research ethics board at Unity Health Toronto, University of Toronto, Canada approved this study. Informed consent was obtained from all participants. Methods were carried out in accordance with the Declaration of Helsinki.^49^

#### Design

This was a ML-based prognostic study using a model development cohort and prospective validation cohort. Findings are reported based on the Transparent Reporting of a multivariable Prediction model for Individual Prognosis or Diagnosis (TRIPOD) statement (Data S1).^50^

#### Cohort

The sample consisted of consecutive patients with and without PAD presenting to vascular surgery clinics at St. Michael’s Hospital, University of Toronto. For the model development cohort, recruitment took place between January 2018 – August 2019. For the prospective validation cohort, recruitment took place between September 2019 – December 2020. PAD was defined as ABI < 0.9 or toe brachial index (TBI) < 0.67 and absent/diminished pedal pulses.^51^ Patients with acute limb ischemia, acute coronary syndrome, or elevated troponin within the past 3 months were excluded to reduce the risk of confounding on biomarker levels. Over the study period, patients received care from the same 5 vascular surgeons at our institution.

#### Demographic and clinical characteristics

Baseline characteristics recorded included age, sex, hypertension (systolic blood pressure ≥ 130 mmHg, diastolic blood pressure ≥ 80 mmHg, or taking antihypertensive therapy), dyslipidemia (total cholesterol > 5.2 mmol/L, triglyceride > 1.7 mmol/L, or taking lipid-lowering therapy), diabetes (hemoglobin A1c ≥ 6.5% or taking an antihyperglycemic), smoking (current or past), Coronary Artery Disease (CAD), Congestive Heart Failure (CHF), previous stroke, and statin use.^52^^,^^53^ Definitions for cardiovascular risk factors were based on American College of Cardiology guidelines.^52^^,^^53^ Information related to patient sex and age can be found in Tables 1 and 4. Information related to ancestry, race, or ethnicity were not recorded in this study.

### Method details

#### Quantification of plasma biomarker levels

From collected patient blood samples, plasma concentrations of 37 proteins were measured in duplicate using a commercially available LUMINEX assay (Bio-Techne, Minneapolis, MN, USA) according to the manufacturer’s instructions.^54^ The following proteins were chosen based on their involvement in various metabolic processes associated with PAD: inflammation [interleukin (IL)-33,^55^ IL-2,^56^ urokinase Plasminogen Activator Receptor (uPAR),^57^ chemokine ligand (CXCL) 1^58^ and 6,^59^ thyroid peroxidase (TPO),^60^ Tumor Necrosis Factor (TNF) Receptor (R) I and II,^61^ Cluster of Differentiation (CD) 40 and CD40 ligand,^62^ TNF Receptor Superfamily (RSF) 9 / CD137,^63^ galectin-1,^64^ cathepsin S,^65^ A Proliferation-Inducing Ligand (APRIL) / TNFSF13,^66^ Activated Leukocyte Cell Adhesion Molecule {ALCAM/CD166),^67^ Epithelial Cell Adhesion Molecule (EpCAM/TROP1),^68^ alpha 1-microglobulin^7^], Endothelial Dysfunction [endothelial cell-specific Molecule (ESM)-1,^69^ TNF-Stimulated Gene (TSG)-14,^70^ P-selectin (CD62P),^71^ Tyrosine-protein kinase receptor (Tie-2),^72^ angiopoietin-1^73^], angiogenesis [Stem Cell Factor (SCF),^74^ Bone Morphogenetic Protein (BMP)-2,^75^ cytokine nicotinamide Phosphoribosyltransferase (PBEF/visfatin),^76^ endoglin/CD105,^77^ endostatin^78^], atherosclerosis [Proprotein Convertase Subtilisin/Kexin type 9 (PCSK9),^79^ Angiopoietin-like protein 3 (ANGPTL3),^80^ aggrecan,^81^ Growth Differentiation Factor (GDF) 15^8^,], metabolic syndrome [Receptor for Advanced Glycation End proteins (RAGE),^82^ ANGPTL6, chemerin^83^], thrombosis [A Disintegrin And Metalloproteinase with a Thrombospondin type 1 motif, member 13 (ADAMTS13),^84^ Intercellular Adhesion Molecule (ICAM)-1^5^], and renal dysfunction [Kidney Injury Molecule (KIM)-1^85^]. These proteins were selected based on previous literature demonstrating their association with PAD prognosis.^10^^,^^11^^,^^14^ Prior to sample analysis, Fluidics Verification and Calibration bead kits (Luminex Corp)^86^ were used to calibrate the MagPix analyzer (Luminex Corp; Austin, Texas).^87^ At least 50 beads for each protein were acquired and analyzed using Luminex xPonent software.^88^

#### Outcomes

Outpatient clinic visits were performed at 1-year and 2-years following baseline assessment. The primary outcome was 2-year Major Adverse Limb Events (MALE) defined as need for vascular intervention (open or endovascular lower extremity revascularization), or major lower extremity amputation (above the ankle). The individual components of MALE were also investigated. The secondary outcome was 2-year worsening PAD, defined as ABI drop ≥ 0.15, which has previously been verified to be clinically relevant.^89^^,^^90^^,^^91^ Preliminary analysis demonstrated that all adverse events occurred in patients with PAD; therefore, prognostic models for predicting adverse events were built only on the PAD cohort.

#### Model development

Proteins which were differentially expressed in patients with vs. without PAD and were used as predictive features to build a random forest ML model for PAD prognostication. Random forest is an ensemble learning method that operates through multiple decision trees,^92^ which classify populations into branch-like segments to develop prediction algorithms for a target outcome using multiple covariates.^93^ Given its non-parametric nature, random forest can efficiently handle large and complex datasets.^93^ This ML algorithm, which is widely used in literature, demonstrates excellent performance for predicting health outcomes.^94^^,^^95^^,^^96^

Data from the model development cohort was randomly split into training (70%) and test (30%) sets, which is a common method for training and evaluating ML algorithms.^97^ Then, the models were trained with the significantly differentially expressed biomarkers between PAD and non-PAD as input features. The most important predictive features was determined by calculating the variable importance score (gain), a measure of the relative impact of individual covariates in contributing to an overall prediction.^98^ Each model then underwent 10-fold cross-validation, a commonly used methodology to iteratively test a ML model’s ability to make predictions on new data that was not used to train the algorithm.^99^ This method also helps to address outcome imbalance issues.^99^ Next, the model was evaluated on unseen test data and further assessed on the prospective validation cohort with the addition of demographic and clinical characteristics as input features.

### Quantification and statistical analysis

In the model development cohort, optimal propensity-score matching with no replacement was performed to match PAD and non-PAD patients based on all recorded baseline characteristics including age, sex, and comorbidities. Propensity scores were generated for each covariate using log-odds, and groups were matched on these propensity scores using a calibration of 0.1 absolute units. Baseline differences between groups for continuous variables were calculated using independent t-test (if normally distributed) or Mann-Whitney U test (if non-normally distributed). Chi-square test was used to compare categorical variables. Two-year event rates were compared between PAD and non-PAD patients using chi-square test. Protein levels were compared between patients with vs. without PAD using independent t-test (if normally distributed) or Mann-Whitney U test (if non-normally distributed). Predictive ability of the model was assessed for PAD prognosis in the PAD cohort (predicting 2-year MALE, vascular intervention, and worsening PAD status) given that all adverse limb events occurred in the PAD cohort. Supplemental analysis of generalizability of prognostic performance in the overall population was conducted on both PAD and non-PAD patients. Diagnostic performance for discriminating PAD and non-PAD patients was also assessed in the supplemental analysis. The primary metric for assessing model performance was AUROC, a validated metric to assess discriminatory ability that considers both sensitivity and specificity.^100^ Statistical analysis was performed separately on the model development and prospective validation cohorts. Significance was set at a two-tailed p < 0.05. All analyses were carried out using SPSS software version 23 (SPSS Inc., Chicago, Illinois, USA).^101^

## Supplemental information

Document S1. Figures S1–S3

Data S1. Transparent Reporting of a Multivariable Prediction Model for Individual Prognosis or Diagnosis (TRIPOD) Checklist for Prediction Model Development, related to STAR Methods, Design
