# Supplementary material for: A machine learning algorithm for peripheral artery disease prognosis using biomarker data
Source: iScience. 2024 Feb 1;27(3):109081. doi: 10.1016/j.isci.2024.109081 (PMC10867451; doi:10.1016/j.isci.2024.109081)
Supplement: Document S1. Figures S1–S3 [file mmc1.pdf]

iScience, Volume *27*

Supplemental information

A machine learning algorithm for peripheral artery disease prognosis using biomarker data

Ben Li, Farah Shaikh, Abdelrahman Zamzam, Muzammil H. Syed, Rawand Abdin, and Mohammad Qadura

A.) 2-Year MALE

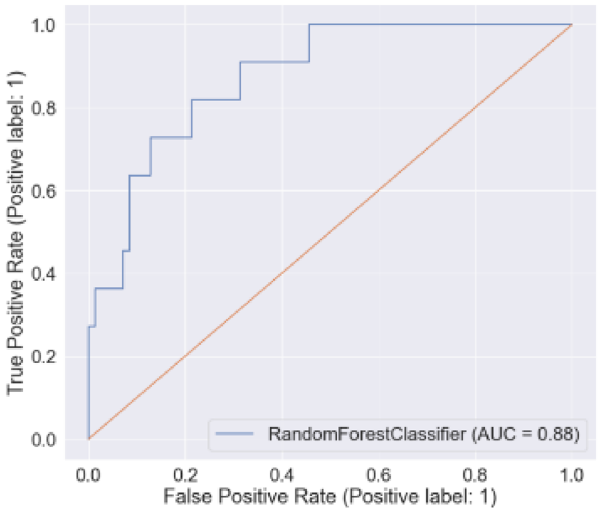

B.) 2-Year vascular intervention

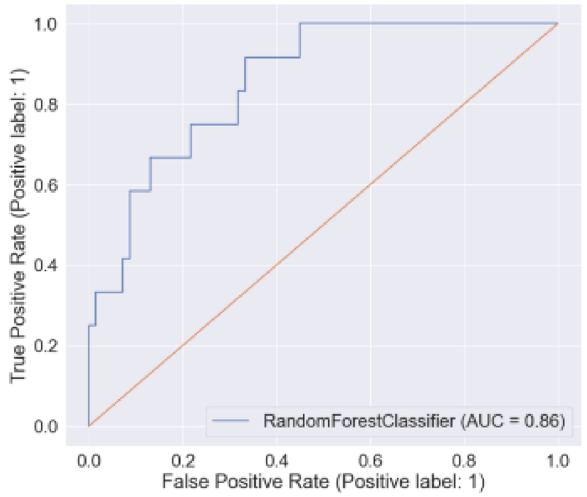

C.) 2-Year worsening PAD status

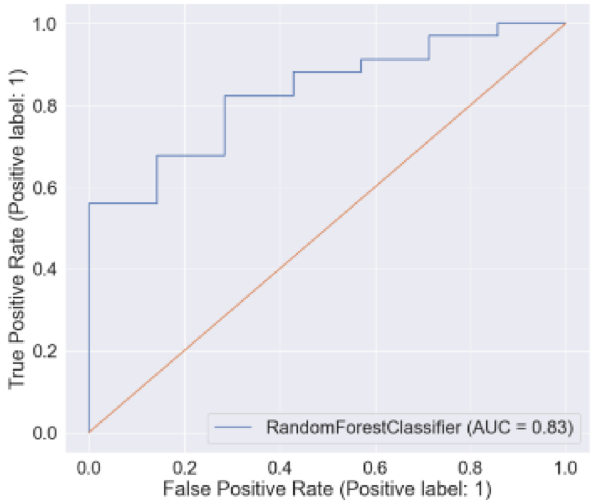

Figure S1. Receiver operating characteristic curve for random forest machine learning model in predicting primary and secondary outcomes on test set data for both PAD and non-PAD patients, related to Figure 1. A.) 2-Year major adverse limb event (MALE). B.) 2-Year vascular intervention. C.) 2-Year worsening PAD status.

Abbreviations: AUC (area under the receiver operating characteristic curve), PAD (peripheral artery disease).

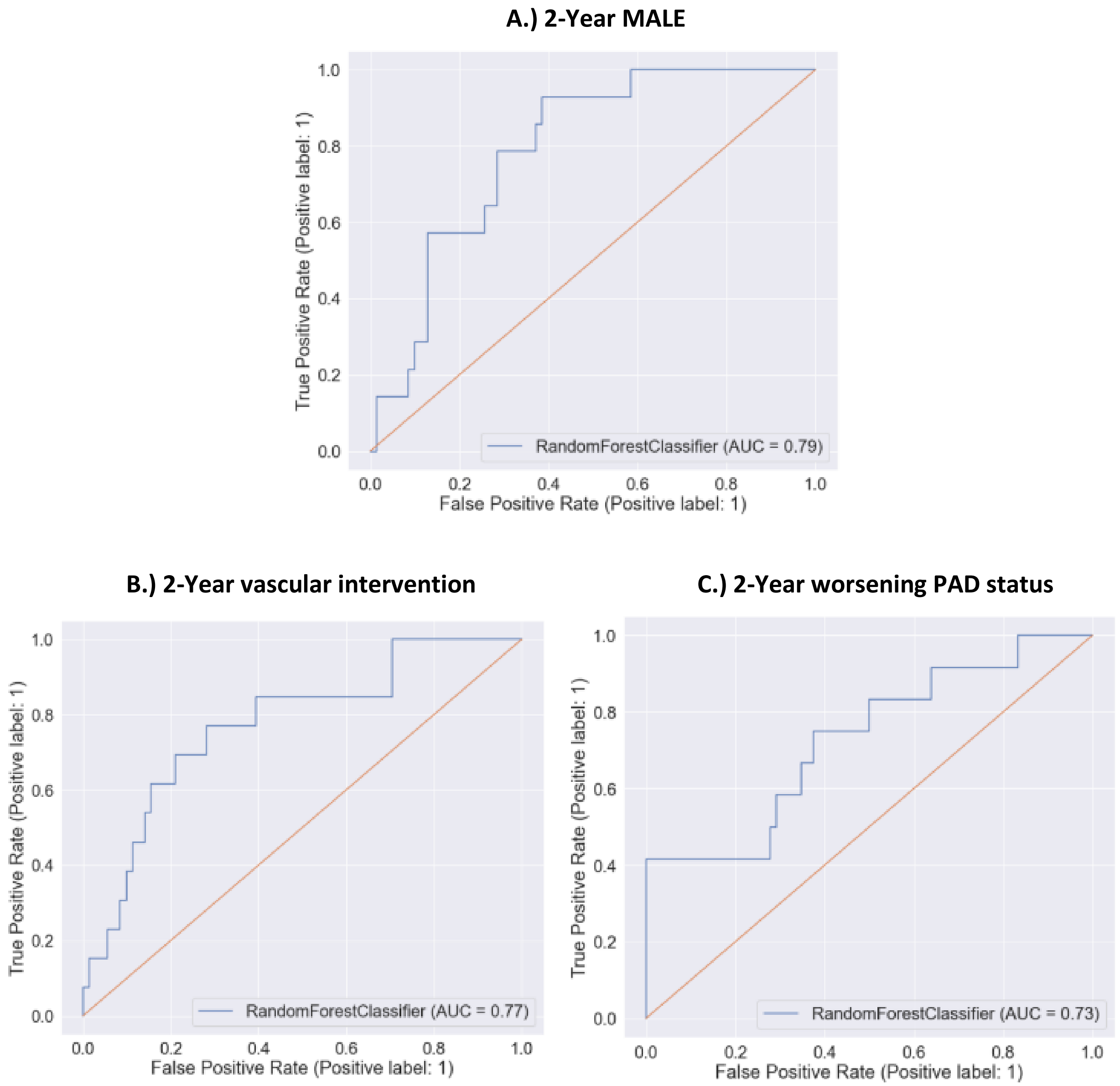

Figure S2. Receiver operating characteristic curve for random forest machine learning model in predicting primary and secondary outcomes in prospective validation cohort for both PAD and non-PAD patients, related to Figure 3. A.) 2-Year major adverse limb event (MALE). B.) 2-Year vascular intervention. C.) 2-Year worsening PAD status. Abbreviations: AUC (area under the receiver operating characteristic curve), PAD (peripheral artery disease).

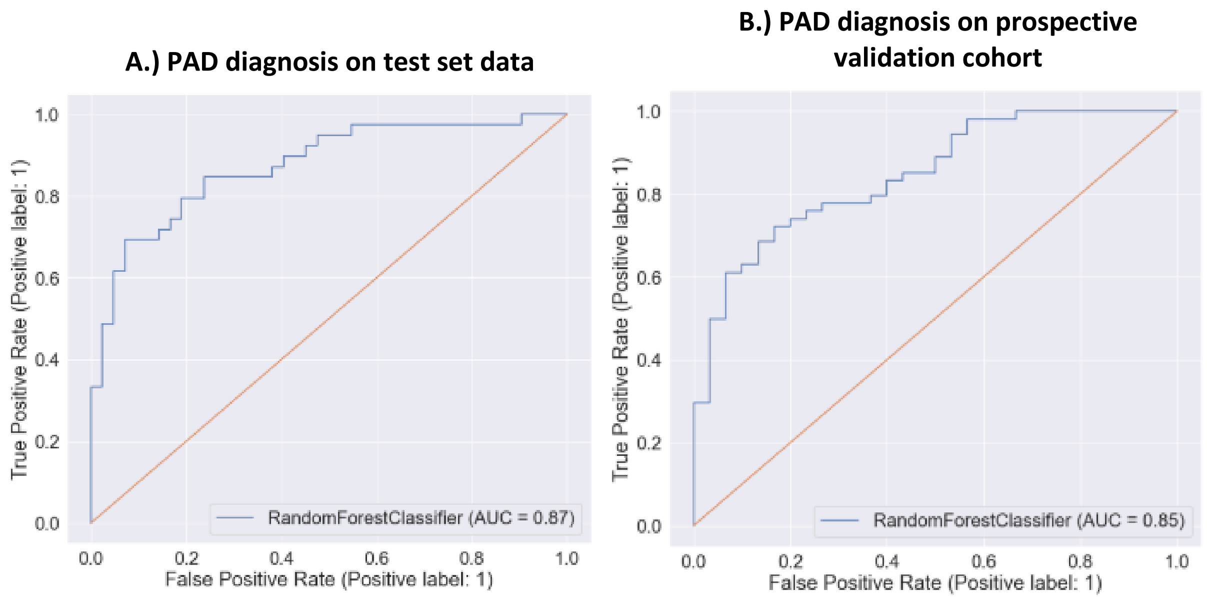

Figure S3. Receiver operating characteristic curve for random forest machine learning model in predicting PAD diagnosis, related to Figures 1 and 3. A.) Test set data. B.) Prospective validation cohort.

Abbreviations: AUC (area under the receiver operating characteristic curve), PAD (peripheral artery disease).
